# Supplementary material for: Rational Design for the Complete Synthesis of Stevioside in Saccharomyces cerevisiae
Source: Microorganisms. 2024 May 31;12(6):1125. doi: 10.3390/microorganisms12061125 (PMC11206123; doi:10.3390/microorganisms12061125)
Supplement: Supplementary file 1 [file microorganisms-12-01125-s001.zip › microorganisms-3037919-supplementary.pdf]

Supplementary Materials

Complete biosynthesis of the natural sweeteners stevioside in Saccharomyces cerevisiae

Wei Huang1, Yongheng Liu1, Xiaomei Ma, Cilang Ma, Yuting Jiang and Jianyu Su *

School of Life Science, Ning Xia University, YinChuan 750000, China

This Supplementary Materials includes:

Table S1. The strain used in this study.

Table S2. The plasmids used in this study.

Table S3. The primers used in this study.

Table S4. The primers used for detection in this study.

Figure S1. Schematic diagram of homologous recombinant plasmid operation

Figure S2. Homologous sequence alignment of different sources of diterpene synthase.

Figure S3. The Liquid chromatogram of fermentation product of recombinant strain.

Table S1. The strain used in this study.

**Strains**	**Description**	**Sources**
E.coli TOP10	F- mcrAΔ(mrr-hsd RMS-mcrBC), φ80, lacZΔM15, ΔlacX74, nupG, recA1, araD139Δ(ara-leu)7696, galK16, rpsL(StrR), endA1	In Our Lab
Saccharomyces cerevisiae BY4742	MAT*α*, *HIS3*Δ, *LEU2*Δ, *LYS2*Δ, *URA3*Δ	In Our Lab
SST-001	BY4742 1309a::P$_{TEF2}$-*SrKS*-T$_{CYC1}$-P$_{PGK1}$-*SrCPS*-T$_{ADH1}$; *YPL062w*::P$_{ENO2}$-*SrKO*-T$_{CYC1}$-P$_{TDH3}$-*SrKAH-SrCPR*-T$_{PGK1}$	This study
SST-101	SST-001 P$_{erg9}$::*Leu2-tHMG1*-P$_{HXT1}$	This study
SST-201	SST-101 *YJL064w*::*His3-tHMG1-UCP2-1*	This study
SST-301/SST-301 Ⅰ	SST-201 *YPLdelta15*::*LoxP*-P$_{ENO2}$-m*erg20~erg20*-T$_{CYC1}$-P$_{TDH3}$-*IDI1*-T$_{PGK1}$	This study
SST-301 Ⅱ	SST-301 1309a::*LoxP*-T$_{PGK1}$-*tHMG1*-P$_{TDH3}$-T$_{ADH1}$-*SrCPS*-P$_{PGK1}$-P$_{TEF2}$ -*SrKS*-T$_{CYC1}$	This study
SST-301Ⅲ	SST-301 1309a::*LoxP*-T$_{PGK1}$-*tHMG1*-P$_{TDH3}$-P$_{PGK1}$-SrKS~SrCPS-T$_{CYC1}$	This study
SST-302 Ⅰ	SST-301 1309a::*LoxP*-T$_{PGK1}$-*tHMG1*-P$_{TDH}$3-T$_{ADH1}$-*SrKS*-P$_{PGK1}$-P$_{TEF2}$ -*AtCPS*-T$_{CYC1}$	This study
SST-302 Ⅱ	SST-301 1309a::*LoxP*-T$_{PGK1}$-*tHMG1*-P$_{TDH3}$-P$_{PGK1}$-*SrCPS-AtKS*-T$_{CYC1}$	This study
SST-302Ⅲ	SST-301 1309a::*LoxP*-T$_{PGK1}$-*tHMG1*-P$_{TDH3}$-P$_{PGK1}$-*AtKS-SrCPS*-T$_{CYC1}$	This study
SST-303 Ⅰ	SST-301 1309a::*LoxP*-T$_{PGK1}$-*tHMG1*-P$_{TDH3}$-T$_{ADH1}$-*ZmKS*-P$_{PGK1}$-P$_{TEF2}$ -*SrCPS*-T$_{CYC1}$	This study
SST-303 Ⅱ	SST-301 1309a::*LoxP*-T$_{PGK1}$-*tHMG1*-P$_{TDH3}$-P$_{PGK1}$-*SrCPS-ZmKS*-T$_{CYC1}$	This study
SST-303Ⅲ	SST-301 1309a::*LoxP*-T$_{PGK1}$-*tHMG1*-P$_{TDH3}$-P$_{PGK1}$*ZmKS-SrCPS*-T$_{CYC1}$	This study
SST-304 Ⅰ	SST-301 1309a::*LoxP*-T$_{PGK1}$-*tHMG1*-P$_{TDH3}$-T$_{ADH1}$-*SrCPS*-P$_{PGK1}$-P$_{TEF2}$ -*SrKS*-T$_{CYC1}$	This study
SST-304 Ⅱ	SST-301 1309a::*LoxP*-T$_{PGK1}$-*tHMG1*-P$_{TDH3}$-P$_{PGK1}$-*AtCPS-SrKS*-T$_{CYC1}$	This study
SST-304Ⅲ	SST-301 1309a::*LoxP*-T$_{PGK1}$-*tHMG1*-P$_{TDH3}$-P$_{PGK1}$-*SrKS-AtCPS*-T$_{CYC1}$	This study
SST-305 Ⅰ	SST-301 1309a::*LoxP*-T$_{PGK1}$-*tHMG1*-P$_{TDH3}$-T$_{ADH1}$-*AtCPS*-P$_{PGK1}$-P$_{TEF2}$ -*AtKS*-T$_{CYC1}$	This study
SST-305 Ⅱ	SST-301 1309a::LoxP-T$_{PGK1}$-*tHMG1*-P$_{TDH3}$-P$_{PGK1}$-*AtCPS-AtKS*-T$_{CYC1}$	This study
SST-305Ⅲ	SST-301 1309a::*LoxP*-T$_{PGK1}$-*tHMG1*-P$_{TDH3}$-P$_{PGK1}$-*AtKS-AtCPS*-T$_{CYC1}$	This study
SST-306 Ⅰ	SST-301 1309a::*LoxP*-T$_{PGK1}$-*tHMG1*-P$_{TDH3}$-T$_{ADH1}$-*AtCPS*-P$_{PGK1}$-P$_{TEF2}$ -*ZmKS*-T$_{CYC1}$	This study
SST-306 Ⅱ	SST-301 1309a::*LoxP*-T$_{PGK1}$-*tHMG1*-P$_{TDH3}$-P$_{PGK1}$-*AtCPS-ZmKS*-T$_{CYC1}$	This study
SST-306Ⅲ	SST-301 1309a::*LoxP*-T$_{PGK1}$-*tHMG1*-P$_{TDH3}$-P$_{PGK1}$-*ZmKS-AtCPS*--T$_{CYC1}$	This study
SST-307 Ⅰ	SST-301 1309a::*LoxP*-T$_{PGK1}$-*tHMG1*-P$_{TDH3}$-T$_{ADH1}$-*ZmtCPS*-P$_{PGK1}$-P$_{TEF2}$ -*SrKS*-T$_{CYC1}$	This study
SST-307 Ⅱ	SST-301 1309a::*LoxP*-T$_{PGK1}$-*tHMG1*-P$_{TDH3}$-P$_{PGK1}$-*ZmtCPS-SrKS*-T$_{CYC1}$	This study
SST-307Ⅲ	SST-301 1309a::*LoxP*-T$_{PGK1}$-*tHMG1*-P$_{TDH3}$-P$_{PGK1}$-*SrKS-ZmtCPS*-T$_{CYC1}$	This study
SST-308 Ⅰ	SST-301 1309a::*LoxP*-T$_{PGK1}$-*tHMG1*-P$_{TDH3}$-T$_{ADH1}$-*ZmtCPS*-P$_{PGK1}$-P$_{TEF2}$ -*AtKS*-T$_{CYC1}$	This study
SST-308 Ⅱ	SST-301 1309a::*LoxP*-T$_{PGK1}$-*tHMG1*-P$_{TDH3}$-P$_{PGK1}$-*ZmtCPS-AtKS*-T$_{CYC1t}$	This study
SST-308Ⅲ	SST-301 1309a::*LoxP*-T$_{PGK1}$-*tHMG1*-P$_{TDH3}$-P$_{PGK1}$-*AtKS-ZmtCPS*-T$_{CYC1t}$	This study
SST-309 Ⅰ	SST-301 1309a::*LoxP*-T$_{PGK1}$-*tHMG1*-P$_{TDH3}$-T$_{ADH1}$-*ZmtCPS*-P$_{PGK1}$-P$_{TEF2}$ -*ZmKS*-T$_{CYC1}$	This study
SST-309 Ⅱ	SST-301 1309a::*LoxP*-T$_{PGK1}$-*tHMG1*-P$_{TDH3}$-P$_{PGK1}$-*ZmtCPS-ZmKS*-T$_{CYC1}$	This study
SST-309Ⅲ	SST-301 1309a::*LoxP*-T$_{PGK1}$-*tHMG1*-P$_{TDH3}$-P$_{PGK1}$-*ZmKS-ZmtCPS*-T$_{CYC1}$	This study

SST-310Ⅳ	SST-013 1309a::*LoxP*-T$_{PGK1}$-*tHMG1*-P$_{TDH3}$-P$_{TEF2}$-*GfKS*-T$_{CYC1}$	This study
SST-311Ⅳ	SST-013 1309a::*LoxP*-T$_{PGK1}$-*tHMG1*-P$_{TDH3}$-P$_{TEF2}$-*PpKS*-T$_{CYC1}$	This study
SST-312Ⅳ	SST-013 1309a::*LoxP*-T$_{PGK1}$-*tHMG1*-P$_{TDH3}$-P$_{TEF2}$-*AgKS*-T$_{CYC1}$	This study
SST-302Ⅲ-ST	SST-302Ⅲ 1414a::*LoxP*-P$_{TEF2}$-*SrUGT85C2*-T$_{CYC1}$-P$_{PGK1}$-*SrUGT74G1*-T$_{ADH1}$-P$_{TDH3}$-*SrUGT91D2e*-T$_{PGK1}$	This study
SST-302Ⅲ-ST1	SST-302Ⅲ-ST 1414a::*LoxP*-P$_{PGK1}$-*SrUGT74G1*$_{S84A/E87A}$-*SrUGT85C2*--T$_{PGK1}$-P$_{TEF2}$-*SrUGT91D2e_NO.5*-T$_{ADH1}$	This study
SST-302Ⅲ-ST2	SST-302Ⅲ-ST1 1622b:: *LoxP*-P$_{TEF2}$-*HXK1*-T$_{TEF2}$-P$_{PGK1}$-*PGM2*-T$_{ADH1}$-P$_{TDH3}$-*UGP1*-T$_{PGK1}$	This study

Table S2. The plasmids used and constructed in this study.

Plasmid	Description	Sources
pRS426	2μ, *URA3*, *AmpR*	In our Lab
pUC57-KanR-*SrCPS*	Synthetic *SrCPS*	This study
pUC57-KanR-*SrKS*	Synthetic *SrKS*	This study
pUC57-KanR-*SrKO*	Synthetic *SrKO*	This study
pUC57-KanR-*SrKAH-CPR*	Synthetic *SrKAH-CPR*	This study
pUC57-KanR-*SrUGT85C2*	Synthetic *SrUGT85C2*	This study
pUC57-KanR-*SrUGT91D2*	Synthetic *SrUGT91D2*	This study
pUC57-KanR-*SrUGT74G1*	Synthetic *SrUGT74G1*	This study
pUC57-KanR-*AtCPS*	Synthetic *AtCPS*	This study
pUC58-KanR-*AtKS*	Synthetic *AtKS*	This study
pUC58-KanR-*ZmtCPS*	Synthetic *ZmtCPS*	This study
pUC57-KanR-*ZmKS*	Synthetic *ZmKS*	This study
pUC57-KanR-*GfKS*	Synthetic *GfKS*	This study
pUC57-KanR-*PpKS*	Synthetic *PpKS*	This study
pUC57-KanR-*AgAS*	Synthetic *AgAS*	This study
pUC57-KanR-*UCP2-1*	Synthetic the mutant of *UCP*	This study
pUC57-KanR-*SrUGT91D2e_NO.5*	Synthetic *SrUGT91D2e_NO.5*	This study
pUC57-Lte9	pUC possessing HU-P_{LEU2}-*LEU2*-T_{LEU2}-P_{TEF1}-*tHMG1*-T_{ADH1}-P_{HXT1}-HD	This study
pUC57-HtU	pUC possessing HU-P_{HIS3}-*HIS3*-T_{HIS3}-P_{TEF1}-*tHMG1*-T_{ADH1}-P_{ENO2}-*UCP2-1*-T_{CYC1}-UD	This study
pUSC01	*LoxP-KanR-ori-URA3-LoxP*-T_{PGK1}-P_{TDH3}-P_{ENO2}-T_{CYC1}	This study
pUSC02	*LoxP-KanR-ori-URA3-LoxP*-T_{ADH1}-P_{PGK1}-P_{TEF2}-T_{TEF2}	This study
pUSC-mEI	pUSC01 possessing HU-T_{CYC1}-*mERG20-ERG20*-P_{ENO2}-P_{TDH3}-*IDI1*-T_{PGK1}	This study
p1309CK(pUt301I)	pUSC02 possessing HU-T_{PGK1}-*tHMG1*-P_{TDH3}-T_{ADH1}-*SrCPS*-P_{PGK1}-P_{TEF2} -*SrKS*-T_{CYC1}	This study
pYPL	pUSC01 possessing HU- T_{CYC1}-*SrKO*-P_{ENO1}-P_{TDH3}-*SrKAH-SrCPR*-T_{PGK1}	This study
pUt301 II	pUSC01 possessing HU-T_{PGK1}-*tHMG1*-P_{TDH3}-P_{PGK1}-*SrCPS*~*SrKS*-T_{CYC1}	This study
pUt301III	pUSC01 possessing HU-T_{PGK1}-*tHMG1*-P_{TDH3}-P_{PGK1}-SrKS~SrCPS-T_{CYC1}	This study
pUt302I	pUSC01 possessing HU-T_{PGK1}-*tHMG1*-P_{TDH}3-T_{ADH1}-*SrCPS*-P_{PGK1}-P_{TEF2} -*AtKS*-T_{CYC1}	This study
pUt302 II	pUSC01 possessing HU-T_{PGK1}-*tHMG1*-P_{TDH3}-P_{PGK1}-*SrCPS-AtKS*-T_{CYC1}	This study
pUt302III	pUSC01 possessing HU-T_{PGK1}-*tHMG1*-P_{TDH3}-P_{PGK1}-*AtKS-SrCPS*-T_{CYC1}	This study

pUt303I	pUSC01 possessing HU-T_{PGK1}-*tHMG1*-P_{TDH3}-T_{ADH1}-*SrCPS*-P_{PGK1}-P_{TEF2} -*ZmKS*-T_{CYC1}	This study
pUt303 II	pUSC01 possessing HU-T_{PGK1}-*tHMG1*-P_{TDH3}-P_{PGK1}-*SrCPS*-*ZmKS*-T_{CYC1}	This study
pUt303III	pUSC01 possessing HU-T_{PGK1}-*tHMG1*-P_{TDH3}-P_{PGK1}-*ZmKS*-*SrCPS*-T_{CYC1}	This study
pUt304I	pUSC01 possessing HU-T_{PGK1}-*tHMG1*-P_{TDH3}-T_{ADH1}-*SrCPS*-P_{PGK1}-P_{TEF2} -*SrKS*-T_{CYC1}	This study
pUt304 II	pUSC01 possessing HU-T_{PGK1}-*tHMG1*-P_{TDH3}-P_{PGK1}-*AtCPS*-*SrKS*-T_{CYC1}	This study
pUt304III	pUSC01 possessing HU-T_{PGK1}-*tHMG1*-P_{TDH3}-P_{PGK1}-*SrKS*-*AtCPS*-T_{CYC1}	This study
pUt305I	pUSC01 possessing HU-T_{PGK1}-*tHMG1*-P_{TDH3}-T_{ADH1}-*AtCPS*-P_{PGK1}-P_{TEF2} -*AtKS*-T_{CYC1}	This study
pUt305 II	pUSC01 possessing HU-T_{PGK1}-*tHMG1*-P_{TDH3}-P_{PGK1}-*AtCPS*-*AtKS*-T_{CYC1}	This study
pUt305III	pUSC01 possessing HU-T_{PGK1}-*tHMG1*-P_{TDH3}-P_{PGK1}-*AtKS*-*AtCPS*-T_{CYC1}	This study
pUt306I	pUSC01 possessing HU-T_{PGK1}-*tHMG1*-P_{TDH3}-T_{ADH1}-*AtCPS*-P_{PGK1}-P_{TEF2} -*ZmKS*-T_{CYC1}	This study
pUt306 II	pUSC01 possessing HU-T_{PGK1}-*tHMG1*-P_{TDH3}-P_{PGK1}-*AtCPS*-*ZmKS*-T_{CYC1}	This study
pUt306III	pUSC01 possessing HU-T_{PGK1}-*tHMG1*-P_{TDH3}-P_{PGK1}-*ZmKS*-*AtCPS*--T_{CYC1}	This study
pUt307I	pUSC01 possessing HU-T_{PGK1}-*tHMG1*-P_{TDH3}-T_{ADH1}-*ZmtCPS*-P_{PGK1}-P_{TEF2} -*SrKS*-T_{CYC1}	This study
pUt307 II	pUSC01 possessing HU-T_{PGK1}-*tHMG1*-P_{TDH3}-P_{PGK1}-*ZmtCPS*-*SrKS*-T_{CYC1}	This study
pUt307III	pUSC01 possessing HU-T_{PGK1}-*tHMG1*-P_{TDH3}-P_{PGK1}-*SrKS*-*ZmtCPS*-T_{CYC1}	This study
pUt308I	pUSC01 possessing HU-T_{PGK1}-*tHMG1*-P_{TDH3}-T_{ADH1}-*ZmtCPS*-P_{PGK1}-P_{TEF2} -*AtKS*-T_{CYC1}	This study
pUt308 II	pUSC01 possessing HU-T_{PGK1}-*tHMG1*-P_{TDH3}-P_{PGK1}-*ZmtCPS*-*AtKS*-T_{CYC1t}	This study
pUt308III	pUSC01 possessing HU-T_{PGK1}-*tHMG1*-P_{TDH3}-P_{PGK1}-*AtKS*-*ZmtCPS*-T_{CYC1t}	This study
pUt309I	pUSC01 possessing HU-T_{PGK1}-*tHMG1*-P_{TDH3}-T_{ADH1}-*ZmtCPS*-P_{PGK1}-P_{TEF2} -*ZmKS*-T_{CYC1}	This study
pUt309 II	pUSC01 possessing HU-T_{PGK1}-*tHMG1*-P_{TDH3}-P_{PGK1}-*ZmtCPS*-*ZmKS*-T_{CYC1}	This study
pUt309III	pUSC01 possessing HU-T_{PGK1}-*tHMG1*-P_{TDH3}-P_{PGK1}-*ZmKS*-*ZmtCPS*-T_{CYC1}	This study
pUt310IV	pUSC01 possessing HU-T_{PGK1}-*tHMG1*-P_{TDH3}-P_{TEF2}-*GfKS*-T_{CYC1}	This study
pUt311IV	pUSC01 possessing HU-T_{PGK1}-*tHMG1*-P_{TDH3}-P_{TEF2}-*PpKS*-T_{CYC1}	This study
pUt312IV	pUSC01 possessing HU-T_{PGK1}-*tHMG1*-P_{TDH3}-P_{TEF2}-*AgKS*-T_{CYC1}	This study

pU-302III-ST	pUSC01 possessing HU-P_{TEF2}-*SrUGT85C2*-T_{CYC1}-P_{PGK1}-*SrUGT74G1*-T_{ADH1}-P_{TDH3}-*SrUGT91D2e*-T_{PGK1}-HD	This study
pU-302III-ST1	pUSC01 possessing HU-P_{PGK1}-*SrUGT74G1*$_{S84A/E87A}$-*SrUGT85C2*-T_{PGK1}-P_{ENO2}-*SrUGT91D2e_NO.5*-T_{ADH1}-HD	This study
pU-302III-ST2	pUSC01 possessing HU-P_{TEF2}-*HXK1*-T_{TEF2}-P_{PGK1}-*PGM2*-T_{ADH1}-P_{TDH3}-*UGP1*-T_{PGK1}-HD	This study

Table S3. The primers used for construction of plasmid in this study.

Primers name	Sequence	size	Description
PUSC01-1F	ACCCTATGCGGTGTGAAATACCGCA	2148bp	pUC57 as the template, amplified ori and *KanR* fragments , and added a codirectional *LoxP* sequence
PUSC01-1R	CCATAACTTCGTATAGCATACATTATACGAAGT TATGCGCGCTCACTGGCCGTCGT		
PUSC01-2F	GCTATACGAAGTTATGGCCATAAAGGCCTAAA AGCCTTCGAGCGTCCCAA	293bp	terminaor *CYC1*，MSC Ⅰ
PUSC01-2R	CGCTCTAGAACTAGTGGATCCCATGTAATTAGT TATGTCAC		
PUSC01-3F	CACTAGTTCTAGAGCGGCCGCATTATTATTGTA TGTTATAGT	553bp	Promoter *ENO2*，MSC Ⅰ
PUSC01-3R	TTGATAATGAACGCGGCGTTATGTCACTAA		
PUSC01-4F	CGCCGCGTTCATTATCAATACTGCCATT	700bp	Promoter *TDH3*，MSC Ⅱ
PUSC01-4R	GCTTATCGATACCGTCGACCTCGAGTTGTTTGT TTATGTGTGTTT		
PUSC01-5F	GACGGTATCGATAAGCTTGATATCGAATTCGTG TTGCTTTCTTATCCGAA	386bp	Terminaor *PGK1* , added a codirectional *LoxP* sequenceMSC Ⅱ
PUSC01-5R	CGTATAGCATACATTATACGAAGTTATAAATA ATATCCTTCTCGAAAGC		
PUSC01-6F	CGTATAATGTATGCTATACGAAGTTATGAACAT GTGAGCAAAAGGCCAGC	1198bp	Use pRS426 as template, amplified *URA3*, and added acodirectional *LoxP* sequence
PUSC01-6R	TTTCACACCGCATAGGGTAATAACTGATATAA		
PUSC02-1F	GGCGTTAAAGCTTTCGAGAAGGAT	3364bp	Use pUSC01 as template, amplified plasmid
PUSC02-1R	AGGCCTTTATGGCCATAACTT		
PUSC02-2F	ATGGCCATAAAGGCCTATGTTGATAGCAGCTTT TT	309bp	Terminaor *TEF2t*, MSC Ⅰ
PUSC02-2R	GCCGCTCTAGAACTAGTGGATCCGAGTAATAA TTATTGCTTCC		
PUSC02-3F	ACTAGTTCTAGAGCGGCCGCTTTAGTTAATTAT AGTTCGT	618bp	Promoter *TEF2*, MSC Ⅰ
PUSC02-3R	TCTTTCCTTACTCACCAATATATATATATATATA T		
PUSC02-4F	GGTGAGTAAGGAAAGAGTGAGG	727bp	Promoter *PGK1*, MSC Ⅱ
PUSC02-4R	CAAGCTTATCGATACCGTCGACCTCGAGTTGTT TTATATTTGTTGTAAA		
PUSC02-5F	CGGTATCGATAAGCTTGATATCGAATTCGCGA ATTTCTTATGATTTAT	234bp	terminaor *ADH1t*, MSC Ⅱ
PUSC02-5R	CGAAAGCTTTAACGCCGGTAGAGGTGTGGTCA AT		
UP13-F	TACGAAGTTATGGCCAATAGTAAGTTTCTTTGA TAC	517bp	Upstream homologous arm of *ARS1309a* site.
UP13-R	TAGGCCTTTATGGCCTAGTCTGGGTTTTTTTCAG		

	T		
DOWN13-F	AGGCCATAAAGGCCTAGCGGCAAAATTAAACGAAAA	547bp	Downstream homologous arm of *ARS1309a* site.
DOWN13-R	TTTTGGGACGCTCGAAGGCTTTTAGGCCTAGGTCTAC		
SrKS001-F	CTTCGAGCGTCCCAAAACCTTCTC	2562bp	Synthetic *SrKS* and its terminator
SrKS001-R	CCGGATGAATTTGTCTTTATGTAT		
Pro001-F	AGACAAATTCATCCGGTTTAGTTAATTATAGTTCGT	1304bp	Bidirectional promotor TEF2 and PGK1
Pro001-R	GAACGGATGATGTTCATTTGTTTTATATTTGTTGTAA		
SrCPS001-F	ATGAACATCATCCGTTCTCCAC	2530bp	Synthetic *SrCPS* and its terminaor
SrCPS001-R	GATAATGACCGGTAGAGGTGTGGTCAATA		
tHMG1001-F	TCTACCGGTCATTATCAATACTGCCATTT	2263bp	tHMG1 and its promoter
tHMG1001-R	CAATTCAATCGCTTAGGATTTAATGCAGG		
P001-F	CCTAAGCGATTGAATTGAATTGAAATCGA	3633bp	Skeleton plasmid fragment, using pUSC02 as template
P001-F	TGGCCATAACTTCGTATAGC		
UPYPL-F	GAAGTTATGGCCATCATTTATGTCGAAA	425bp	Upstream homologous arm of *YPL062w* site.
UPYPL-R	CCTTTATGGCCCATAAAAATCCAAAAAAAAA		
DYPL-F	TTATGGGCCATAAAGGCCGACTGAACACTTCGAATTGA	433bp	Downstream homologous arm of *YPL062w* site
DYPL-R	AGGCTTTTAGGCCTAATAAGGCAGCCGACAAAAG		
SrKOYPL-F	TTAGGCCTAAAAGCCTTCGAGCGTCCCAA	1793bp	Synthetic *SrKO* and its terminaor
SrKOYPL-R	ATGTAGGATGCTGTGACGGGTTT		
ProYPL-F	TCACAGCATCCATCATTATTATTGTATGTTATA	1203bp	Bidirectional promotor *TEF2* and *PGK1*
ProYPL-R	GTTGTTTGTTTATGTGTGTTTATTCGAAACTAAG		
SrKAHYPL-F	CACACATAAACAAACAACATGATACAAGTTTTAACTCCAA	3609bp	Synthetic fused *SrKAH-SrCPR*, and terminaor
SrKAHYPL-R	TTATCGATACCGTCGACCTTACCAAACATCTCTTAAGTAT		
PYPL-F	GGTCGACGGTATCGATAAATTGAATTGAATTGAAATCGAT	3684bp	Skeleton plasmid fragment, using pUSC01 as template
PYPL-R	GATGGCCATAACTTCGTATA		
UPLte9-F	AACTAGTGGATTCCAACTTATTCCCCTGTGCCTA	537bp	Upstream homologous arm of P_{erg9} site.
UPLte9-R	CTGAGTATTCCCACAGTTGATGCAAAGTGCAGTGGAAA		
LeuLte9-F	CTGTGGGAATACTCAGGTATCGTAAG	1997bp	*LEU2* and its promoter and terminaor
LeuLte9-R	TTCTGGGCCTCCATGTCTTTCGACTACGTCGTTAAGGCC		
tHMG1Lte9-F	AAAGACATGGAGGCCCAGAATACC	2113bp	2# tHMG1 with its promoter and terminaor
tHMG1Lte9-R	AAGCCGGTAGAGGTGTGGTCAATAAGAGC		
HXT1pLte9-F	ACACCTCTACCGGCTTGCAGGTCTCATCTGGAATATAA	1144bp	Promoter *HXT1*

HXT1pLte9-R	GCCTTCCGATTTTACGTATATCAACTA		
DLte9-F	CGTAAAATCGGAAGGCGTTATCGGT	555bp	Downstream homologous arm of P_{erg9} site.
DLte9-R	CCTCGAGAATCTGTCAAAACGGCTCTG		
PLte9-F	TTGACAGATTCTCGAGGGGGGGCCCGGTA	2215bp	Skeleton plasmid fragment, using pUC57 as template
PLte9-R	GTTGGATCCACTAGTTCTAGAGCGG		
UPHtU-F	CATGTTCTACAGATCGTGAGAGAG	532bp	Upstream homologous arm of YJL064w site
UPHtU-R	GACTTCGCTCATCATTATTATTGTATGTTATA		
tHMG1HtU-F	GTGGTGGTTCTGACATGGAGGCCCAGAATAC	2125bp	3# tHMG1 with its promoter and terminaor
tHMG1HtU-R	AAAGAAACCGGTAGAGGTGTGGTCAATA		
UCPHtU-F	AATGATGAGCGAAGTCGGTATACA	3509bp	Synthetic *UCP2-1* with its promoter and terminator
UCPHtU-R	AAAAGCCTTCGAGCGTCCCAAA		
DHtU-F	ACGCTCGAAGGCTTTTATTCTCTCTTGTTTCTTCTG	545bp	Downstream homologous arm of *YJL064w* site.
DHtU-R	TGACCATGATTACGCCTAAAGATGTGGCTGTTAGTC		
PHtU-F	GGCGTAATCATGGTCATAGCTGT	2203bp	Skeleton plasmid fragment, using pUC57 as template
PHtU-R	CGATCTGTAGAACATGATAATAATGGTTTCTT		
UPd20-F	TGTCGAAAAACAGGCCATTCTACGTAATATTTTTGAG	463bp	Upstream homologous arm of *YPLdelta15* site.
UPd20-R	AAGGGCCTTTATGGCCTGGAATTTTTCACATGTTTG		
Dd20-F	AGGCCATAAAGGCCCTTGGAGGACCCATTATCGT	455bp	Downstream homologous arm of *YPLdelta15* site.
Dd20-R	GAAGGCTTTAACTAACGCTAATCGATAAA		
EMd20-F	GCGTTAGTTAAAGCCTTCGAGCGTCCCAA	2909bp	Synthetic fude gene *mErg20-Erg20*, and their promoter and terminaor
EMd20-R	GATAATGAACGCGGCGTTATGTCACTAAC		
IDId20-F	ACGCCGCGTTCATTATCAATACTGCCATT	3532bp	*IDI1* with its promoter
IDId20-R	ACGCAATGGAATAGGCTAAGAT		
PEMId20-F	GCCTATTCCATTGCGTACCGCCTCTAATTGAATTGAATTGAAATCGA	3685bp	Skeleton plasmid fragment, using pUSC01 as template
PEMId20-F	TGGCCTGTTTTTCGACATAAATGA		
SrKS-301Ⅱ-F	TTAGAGCGGATGTGGGAGGAGGGCGTGAATGT	2428bp	SrKS, 5' end contain Linker sequence
SrKS-301Ⅱ-R	GGTGGTGGTGGTTCTATGAATTTGTCTTTATGTAT		
SrKCPS-301Ⅱ-F	ATAGAACCACCACCACCCCAAATTACTTTTTGGAACA	3054bp	SrCPS and promoter, 3' end contain Linker sequence
SrCPS-301Ⅱ-R	AATGAGTGAGTAAGGAAAGAGTGAGG		
P1-301Ⅱ-F	CCTTACTCACTCATTATCAATACTGCCATTTC	3759bp	Skeleton plasmid fragment Ⅰ, using pUt-301I as template
P1-301Ⅱ-R	ACCGCATAGGGTAATAACTGATA		
P2-301Ⅱ-F	TATTACCCTATGCGGTGTGAAATA	3377bp	Skeleton plasmid fragment Ⅱ, using pUt-301I as template
P2-301Ⅱ-R	CTCCCACATCCGCTCTAACCGAAAAGGAA		
SrCPS-301Ⅲ-F	GCGTGACATAACTAATTACATGTTACCAAATTACTTTTTGGA	2370bp	*SrCPS*, 5' end contain Linker sequence

SrCPS-301Ⅲ-R	GAGGTGGTGGTTCTATGAACATCATCCGTTCTCC		
SrKS-301Ⅲ-F	ATAGAACCACCACCTCTTTGTTCTTCATTTTCAT	2370bp	*SrKS* , 3'end contain Linker sequence
SrKS-301Ⅲ-R	CCGGATGAATTTGTCTTTATGTAT		
P1-301Ⅲ-F	CATCCGGTTGTTTTATATTTGTTGTAAAAA	4464bp	Skeleton plasmid fragment I, using pUt-301I as template
P1-301Ⅲ-R	ACCGCATAGGGTAATAACTGATA		
P2-301Ⅲ-F	TATTACCCTATGCGGTGTGAAATA	3394bp	Skeleton plasmid fragment Ⅱ, using pUt-301I as template
P2-301Ⅲ-R	TTAGTTATGTCACGCTTACATT		
AtKS-302Ⅰ-F	TTTCGGTTAGAGCGGATGTTAGGTCAAAGATTCCTTTT	2396bp	*AtKS*, 3' end contain Linker sequence
AtKS-302Ⅰ-R	AGGTTTAAGGTGGTGGTTCTATGTCCATTAACTTGAGATC		
P1-302Ⅰ-F	CCACCACCTTAAACCTTTTGGAACAAAAC	5372bp	Skeleton plasmid fragment I, using pUt-303I as template, 5' end contain Linker sequence
P1-302Ⅰ-R	CGCTTAGGATTTAATGCAGGTG		
P2-302Ⅰ-F	GCATTAAATCCTAAGCGATTGAAT	4846bp	Skeleton plasmid fragment Ⅱ, using pUt-301I as template
P2-302Ⅰ-R	CATCCGCTCTAACCGAAAAGGA		
AtKS-302Ⅱ-F	TTAGAGCGGATGTGGGAGGAGGGCGTGAATGT	2441bp	*AtKS*, 5' end contain Linker sequence
AtKS-302Ⅱ-R	AGTAATTTGGGGTGGTGGTGGTTCTATGTCCATTAACTTGAGATC		
P1-302Ⅱ-F	CTCCCACATCCGCTCTAACCGAAAAGGAA	3377bp	Skeleton plasmid fragment I, using pUt-301Ⅱ as template
P1-302Ⅱ-R	TATTACCCTATGCGGTGTGAAATA		
P2-302Ⅱ-F	ACCGCATAGGGTAATAACTGATA	6788bp	Skeleton plasmid fragment Ⅱ, using pUt-301Ⅱ as template
P2-302Ⅱ-R	CACCACCCCAAATTACTTTTGGAACAAAA		
AtKS-302Ⅲ-F	CGGATGATGTTCATAGAACCACCACCACCGGTCAAAGATTCCTTTTGCAA	2382bp	*AtKS*, 3' end contain Linker sequence
AtKS-302Ⅲ-R	GTCCATTAACTTGAGATCTTCT		
SrCPS-302Ⅲ-F	TCTATGAACATCATCCGTTCTCC	4470bp	*SrCPS* with its terminator,and and the UP and Downstream of 1309, and ori 和 KanMX, used pUt-302I as template, 5' end contain Linker sequence
SrCPS-302Ⅲ-R	TATTACCCTATGCGGTGTGAAATA		
P-302Ⅲ-F	ACCGCATAGGGTAATAACTGATA	5707bp	Skeleton plasmid fragment , using pUt-301Ⅲas template
P-302Ⅲ-R	GATCTCAAGTTAATGGACATTTGTTTTATATTTGTTGTAAAAA		
SrCPS-303Ⅰ-F	TTTGATGCTCGATGAGTTTTTCTA	5136bp	*SrCPS* with its terminator, and a bidirectional promotor TEF2 and PGK1,and the UP and Downstream arm of 1309, used pUt-302I as template
SrCPS-303Ⅰ-R	TCACTGGCATAGCCATTTGTTTTATATTTGTTGTAAAA		
ZmKS-303Ⅰ-F	ATGGCTATGCCAGTGAAGCTAA	1777bp	Synthetic *ZmKS*
ZmKS-303Ⅰ-R	TCGCTTAAACATCAGATACCAGTGT		
P-303Ⅰ-F	CTGATGTTAAGCGAATTTCTTATGATTTATGA	5791bp	Skeleton plasmid fragment, using pUt-302I as template
P-303Ⅰ-R	CTCATCGAGCATCAAATGAAACTG		

ZmKS-303 II -F	TTTCGGTTAGAGCGGATGTTAAACATCAGATACCAGTG	1812bp	*ZmKS*, 5' end contains the Linker sequence
ZmKS-303 II -R	AAGGTTTAAGGTGGTGGTTCTATGGCTATGCCAGTGAAGCT		
P1-303 II -F	CCACCACCTTAAACCTTTTGGAACAAAAC	5276bp	Skeleton plasmid fragment I, using pUT-303I as template, 5' end contains Linker sequence
P1-303 II -R	CGCTTAGGATTTAATGCAGGTG		
P2-303 II -F	GCATTAAATCCTAAGCGATTGAAT	4846bp	Skeleton plasmid fragment II, using pUt-303I as template
P2-303 II -R	CATCCGCTCTAACCGAAAAGGA		
ZmS-303III-F	TTCATAGAACCACCACCAACATCAGATACCAGTGTAT	2507bp	ZmKS with promotor, 3' end contains Linker sequence
ZmS-303III-R	GGCAGTATTGATAATGAGTGAGTAAGGAAAGAGTGAG		
P1-303III-F	CTCATTATCAATACTGCCATTTCA	4467bp	Skeleton plasmid fragment I, using pUT-303I as template
P1-303III-R	TATTCTGGGCCTCCATGTCGCTGG		
P2-303III-F	ATGGAGGCCCAGAATACCCTCCTTG	4972bp	Skeleton plasmid fragment II, using pUt-303I as template
P2-303III-R	GGTGGTGGTTCTATGAACATCATCCGTTCTCCAC		
SrKS-304 I -F	CAAGGATGTCCATTAACTTGAGATC	4810bp	*SrKS* with its terminator, and PTDH3 of tHMG1, used pUt-301I as template
SrKS-304 I -R	CAATTCAATCGCTTAGGATTTAAT		
Pro-304 I -F	CATCCGGTTTAGTTAATTATAG	1301bp	Bidirectional promotor PTEF2 and PPGK1
Pro-304 I -R	TAATGGACATCCTTGTTTTATATTTGTTGT		
AtCPS-304 I -F	TTTCGGTTAGAGCGGATGTTAAACCTTTTGGAACAAAA	2439bp	AtCPS with its promotor and terminator, and the UP and Downstream of 1309, used pUt-305I as template
AtCPS-304 I -R	TAACTAAACCGGATGTCTTTGCAATACCATGT		
P-304 I -F	CCTAAGCGATTGAATTGAATTGAA	4837bp	Skeleton plasmid fragment, using pUt-303I as template
P-304 I -R	CATCCGCTCTAACCGAAAAGGA		
AtCPS-304 II -F	ATGGAGTAGTAGACCTCAATATATATATATATATAT	3023bp	*AtCPS* with its promotor, 3' end contains Linker
AtCPS-304 II -R	TCATAGAACCACCACCAACCTTTTGGAACAAAACCT		
SrKS-304 II -F	GGTGGTGGTTCTATGAATTTGTCTTTATGTATTG	4811bp	*SrKS* with its terminator, and PTDH3 of tHMG1, used pUt-301I as template
SrKS-304 II -R	CAATTCAATCGCTTAGGATTTAAT		
P-304 II -F	CCTAAGCGATTGAATTGAATTGAA	4632bp	Skeleton plasmid fragment II, using pUt-303I as template
P-304 II -R	AGGTCTACTACTCCATCGTAAAGC		
AtCPS-304III-F	TGCTCGATGAGTTTTTCTAATCAGT	3976bp	*AtCPS* with its terminator, and the UP and Downstream of 1309, used pUt-304I as template
AtCPS-304III-R	GAGGTGGTGGTTCTATGTCTTTGCAATACCATGTT		
SrKS-304III-F	ATAGAACCACCACCTCTTTGTTCTTCATTTTCATT	3754bp	*SrKS* with its terminator, and P_{TDH3} of tHMG1, used pUt-301III as template
SrKS-304III-R	ACCAATTGGTCCTCGAGTTGTT		
P-304304III-F	CTCGAGGACCAATTGGTGAAAACT	4939bp	Skeleton plasmid fragment , using

P-304304III-R	GAAAAACTCATCGAGCATCAAATGA		pUt-301III as template
AtCPS-305 I -F	TAGGCCTAAAAGCCTTCGAGCG	3915bp	*AtCPR* With its terminator and a bidirectional promotor TEF2 and PGK1
AtCPS-305 I -R	GTTAATGGACATCCTTGTTTTATATTTGTTGTAAA		
AtKS-305 I -F	CAAGGATGTCCATTAACTTGAGATCT	4801bp	*AtKS* with its terminator, and *tHMG1* with P_{TDH3}
AtKS-305 I -R	CGCTTAGGATTTAATGCAGGTG		
P-305 I -F	GCATTAAATCCTAAGCGATTGAATT	4657bp	Skeleton plasmid fragment , using pUt-304 I as template
P-305 I -R	GAAGGCTTTTAGGCCTAGGTCT		
AtKS-305 II -F	AAGGTTGGTGGTGGTTCTATGTCCATTAACTTGAGATC	4823bp	*AtKS* with its terminator, and P_{TDH3} of *tHMG1*, used pUt-305I as template
AtKS-305 II -R	CAATTCAATCGCTTAGGATTTAAT		
AtCPS-305 II -F	ATGGAGTAGTAGACCTCAATATATATATATATATAT	3019bp	*AtCPS* with its promotor, 3' end contains Linker
AtCPS-305 II -R	AGAACCACCACCAACCTTTTGGAACAAAACCTTA		
P-305 II -F	CCTAAGCGATTGAATTGAATTGAA	4632bp	Skeleton plasmid fragment, using pUt-305I as template
P-305 II -R	AGGTCTACTACTCCATCGTAAAGC		
AtKS-305III-F	ACATAGAACCACCACCGGTCAAAGATTCCTTTTGCAA	3757bp	*AtKS* With its terminator and a bidirectional promotor *TEF2* and *PGK1*
AtKS-305III-R	CAATTGGTCCTCGAGTTGTTTG		
AtCPS-305III-F	CAACCATGGGTAAGGAAAAGAC	4769bp	*AtCPS* with its terminator, and the UP and Downstream arm of 1309, used pUt-304III as template
AtCPS-305III-R	GGTGGTGGTTCTATGTCTTTGCAATACCATGT		
P-305III-F	ACTCGAGGACCAATTGGTGAAA	4144bp	Skeleton plasmid fragment, using pUt-304IIIas template
P-305III-R	TCCTTACCCATGGTTGTTTATG		
AtCPS-306 I -F	TAGGCCTAAAAGCCTTCGAGCG	3915bp	*AtCPS* With its terminator and a bidirectional promotor *TEF2* and *PGK1*
AtCPS-306 I -R	TGGCATAGCCATCCTTGTTTTATATTTGTTGTAAA		
ZmKS-306 I -F	CAAGGATGGCTATGCCAGTGAAGCTAAC	4213bp	*ZmKS* with its terminator, and P_{TDH3} of *tHMG1*, used pUt-303I as template
ZmKS-306 I -R	CGCTTAGGATTTAATGCAGGTG		
P-306 I -F	GCATTAAATCCTAAGCGATTGAATT	4657bp	Skeleton plasmid fragment, using pUt-303I as template
P-306 I -R	GAAGGCTTTTAGGCCCTAGGTCT		
AtCPS-306 II -F	AGAACCACCACCAACCTTTTGGAACAAAACCTT	3803bp	*AtCPS* and a bidirectional promotor *TDH3* and *PGK1*,used pUt-304 II as template, 3' end contains linker sequence
AtCPS-306 II -R	AATTGGTCCTCGAGTTGTTTGT		
ZmKS-306 II -F	CCATGGGTAAGGAAAAGACTCA	4078bp	*ZmKS* with its terminator, and the UP and Downstream arm of 1309, used pUt-303 II as template
ZmKS-306 II -R	GTTGGTGGTGGTTCTATGGCTATGCCAGTGAAGCTA		
P-306 II -F	AACTCGAGGACCAATTGGTGAA	4141bp	Skeleton plasmid fragment, using pUt-303 II as template
P-306 II -R	TTTTCCTTACCCATGGTTGTTT		
ZmKS-306III-F	TAGAACCACCACCAACATCAGATACCAGTGTATG	3169bp	*ZmKS* and a bidirectional promotor *TDH3* and *PGK1*,used

ZmKS-306III-R	ACCAATTGGTCCTCGAGTTGTTT		pUt-304 II as template, 3' end contains linker sequence
AtCPS-306III-F	CCGACCATCAAGCATTTTATCCGT	4475bp	*AtCPS* with its terminator, and the UP and Downstream arm of 1309, used pUt-304 II as template
AtCPS-306III-R	GTTGGTGGTGGTTCTATGTCTTTGCAATACCATGTT		
P-306III-F	CTCGAGGACCAATTGGTGAAAACT	4378bp	Skeleton plasmid fragment, using pUt-304 II as template
P-306III-R	AATGCTTGATGGTCGGAAGAGG		
SrKS-307 I -F	CCATCCGGTTTAGTTAATTATAGTTCGTTG	5770bp	*SrKS* and a bidirectional promotor *TEF2* and *PGK1,* and P_{TDH3} of *tHMG1,* used pUt-304 I as template.
SrKS-307 I -R	TGGCATGGATACGGAAATTCTCAA		
ZmtCPS-307 I -F	TCGGTTAGAGCGGATGTTACTTTGCGGCACTTACTG	2509bp	Synthetic *ZmtCPS*
ZmtCPS-307 I -R	TAACTAAACCGGATGGTTTTGTCTTCTTCTTG		
P-307 I -F	TTCCGTATCCATGCCATCCATC	5157bp	Skeleton plasmid fragment, using pUt-304 I as template
P-307 I -R	CATCCGCTCTAACCGAAAAGGA		
ZmtCPS-307 II -F	GAGTAGTAGACCTCAATATATATATATATATATA	3095bp	*ZmtCPS* with its promotor
ZmtCPS-307 II -R	TCATAGAACCACCACCCTTTGCGGCACTTACTGGCTC		
SrKS-307 II -F	GGTGGTGGTTCTATGAATTTGTCTTTATGTATTG	4811bp	*SrKS* with its terminator, and P_{TDH3} of *tHMG1*, used pUt-304I as template
SrKS-307 II -R	CAATTCAATCGCTTAGGATTTAAT		
P-307 II -F	CCTAAGCGATTGAATTGAATTGAA	4635bp	Skeleton plasmid fragment, using pUt-304I as template
P-307 II -R	TTGAGGTCTACTACTCCATCGTAAA		
ZmtCPS-307III-F	GCTCGATGAGTTTTTCTAATCAGT	4049bp	*ZmtCPS* with its terminator, and the UP and Downstream arm of 1309, used pUt-304III as template, 5' end contains linker sequence
ZmtCPS-307III -R	GAAAAACTCATCGAGCATCAAATG		
SrKS-307III-F	CATAGAACCACCACCTCTTTGTTCTTCATTTTCATT	3760bp	*SrKS* and a bidirectional promotor *TDH3* and *PGK1,* 3' end contains linker sequence
SrKS-307III-R	TTTTCACCAATTGGTCCTCGAGTT		
P-307III-F	GGACCAATTGGTGAAAACTGAAGT	4934bp	Skeleton plasmid fragment, using pUt-304III as template
P-307III-R	AGGTGGTGGTTCTATGGTTTTGTCTTCTTCTTGTA		
AtKS-308 I -F	TGGTGAGTAAGGAAAGAGTGAG	3936bp	*AtKS* with its promotor and terminator, and the promotor *TDH3*of *tHMG1*
AtKS-308 I -R	TTTTCACCAATTGGTCCTCGAGTT		
ZmtCPS-308 I -F	GCTCGATGAGTTTTTCTAATCAG	4580bp	*ZmtCPS* with its promotor and terminator, and the UP and Downstream arm of 1309, used pUt-305 I as template
ZmtCPS-308 I -R	CTTTCCTTACTCACCAATATATATATATATATATAT		
P-308 I -F	GGACCAATTGGTGAAAACTGAAGT	4927bp	Skeleton plasmid fragment, using pUt-305 I as template
P-308 I -R	GAAAAACTCATCGAGCATCAAATG		

ZmtCPS-308Ⅱ-F	GCTCGATGAGTTTTTCTAATCAG	4372bp	*ZmtCPS* with its promotor, and the UP and Downstream arm of 1309, used pUt-307Ⅱ as template, 3' end contain Linker sequence
ZmtCPS-308Ⅱ-R	AGGTGGTGGTTCTATGGTTTTGTCTTCTTCTTGTA		
AtKS-308Ⅱ-F	AGGGTGGTGGTTCTATGTCCATTAACTTGAGATCTT	4801bp	*SrKS* with its terminator, and P_{TDH3} of *tHMG1*, used pUt-305Ⅱ as template, 5' end contain Linker sequence
AtKS-308Ⅱ-R	GGATTTAATGCAGGTGACGGACCC		
P-308Ⅱ-F	CACCTGCATTAAATCCTAAGCG	3370bp	Skeleton plasmid fragment, using pUt-305Ⅱ as template
P-308Ⅱ-R	GAAAAACTCATCGAGCATCAAA		
ZmtCPS-308Ⅲ-F	GCTCGATGAGTTTTTCTAATCAGT	4047bp	*ZmtCPS* with its terminator, and the UP and Downstream arm of 1309, used pUt-307Ⅲas template, 5' end contain Linker sequence
ZmtCPS-308Ⅲ-R	GTGGTGGTTCTATGGTTTTGTCTTCTTCTTGTA		
AtKS-308Ⅲ-F	AACCATAGAACCACCACCGGTCAAAGATTCCTTTTGCAAA	3766bp	*AtKS* and a bidirectional promotor *TDH3* and *PGK1*, 3' end contains linker sequence
AtKS-308Ⅲ-R	TTTTCACCAATTGGTCCTCGAGTT		
P-308Ⅲ-F	GGACCAATTGGTGAAAACTGAAGT	4934bp	Skeleton plasmid fragment, using pUt-307Ⅲ as template
P-308Ⅲ-R	GAAAAACTCATCGAGCATCAAATG		
ZmKS-309Ⅰ-F	TGGTGAGTAAGGAAAGAGTGAG	3351bp	*ZmKS* with its promotor and terminator, and the promotor *TDH3* of *tHMG1*
ZmKS-309Ⅰ-R	TTTTCACCAATTGGTCCTCGAGTT		
ZmtCPS-309Ⅰ-F	GCTCGATGAGTTTTTCTAATCAG	4580bp	*ZmtCPS* with its promotor and terminator, and the UP and Downstream arm of 1309, used pUt-307Ⅰ as template
ZmtCPS-309Ⅰ-R	CTTTCCTTACTCACCAATATATATATATATATATATAT		
P-309Ⅰ-F	GGACCAATTGGTGAAAACTGAAGT	4927bp	Skeleton plasmid fragment, using pUt-307Ⅰ as template
P-309Ⅰ-R	GAAAAACTCATCGAGCATCAAATG		
ZmtCPS-309Ⅱ-F	GCTCGATGAGTTTTTCTAATCAG	4372bp	*ZmtCPS* with its promotor, and the UP and Downstream arm of 1309, used pUt-308Ⅱ as template, 3' end contain Linker sequence
ZmtCPS-309Ⅱ-R	ATAGAACCACCACCCTTTGCGGCACTTACTGGCTC		
ZmKS-309Ⅱ-F	AAGGGTGGTGGTTCTATGGCTATGCCAGTGAAGCTA	4217bp	*ZmKS* with its terminator, and P_{TDH3} of *tHMG1*, used pUt-306Ⅱ as template, 5' end contain Linker sequence
ZmKS-309Ⅱ-R	GGATTTAATGCAGGTGACGGACCC		
P-309Ⅱ-F	CACCTGCATTAAATCCTAAGCG	3370bp	Skeleton plasmid fragment, using pUt-308Ⅱ as template
P-309Ⅱ-R	GAAAAACTCATCGAGCATCAAATG		
ZmtCPS-309Ⅲ-F	GCTCGATGAGTTTTTCTAATCAGT	4047bp	*ZmtCPS* with its terminator, and the UP and Downstream arm of 1309, used pUt-308Ⅲas template, 5' end contain Linker sequence
ZmtCPS-309Ⅲ-R	GTGGTGGTTCTATGGTTTTGTCTTCTTCTTGTA		
ZmKS-309Ⅲ-F	AACCATAGAACCACCACCAACATCAGATACCAGTGTATGA	3181bp	*ZmKS* and a bidirectional promotor *TDH3* and *PGK1*, 3' end contains linker sequence
ZmKS-309Ⅲ-R	TTTTCACCAATTGGTCCTGAGTT		
P-309Ⅲ-F	GGACCAATTGGTGAAAACTGAAGT	4934bp	Skeleton plasmid fragment, using

P-309III-R	GAAAAACTCATCGAGCATCAAATG		pUt-308III as template
GfKS-310Ⅳ-F	TTCGGTTAGAGCGGATTTACTTCATAGAGGATGACA	2876bp	Sythetic *GfKS*
GfKS-310Ⅳ-R	CATGCCTGGTAAAATTGAAAAT		
Pro-310Ⅳ-F	CAATTTTACCAFFCATGTTTAGTTAATTATAGTTCGT	1314bp	A bidirectional promotor *TEF2* and *PGK1*
Pro-310Ⅳ-R	GTTTTCACCAATTGGTCTTGTTTTATATTTGTTGTAA		
P1-310Ⅳ-F	TATTACCCTATGCGGTGTGAAATA	3344bp	Skeleton plasmid fragment Ⅰ, using pUt-301 Ⅰ as template
P1-310Ⅳ-R	ATCCGCTCTAACCGAAAAGGAAGG		
P2-310Ⅳ-F	GACCAATTGGTGAAAACTGAAGTC	3077bp	Skeleton plasmid fragment Ⅱ, using pUt-301 Ⅰ as template
P2-310Ⅳ-R	ACCGCATAGGGTAATAACTGATA		
PpKS-311Ⅳ-F	TTCGGTTAGAGCGGATTTATTCTGGAACTGGTTCGA	2668bp	Synthetic *PpKS*
PpKS-311Ⅳ-R	CTAAACATGGCTTCTTCTACCTTGATTC		
P1-311Ⅳ-F	TATTACCCTATGCGGTGTGAAATA	3344bp	Skeleton plasmid fragment Ⅰ, using pUt-310Ⅳ as template
P1-311Ⅳ-R	ATCCGCTCTAACCGAAAAGGAAGG		
P2-311Ⅳ-F	GAAGAAGCCATGTTTAGTTAATTATAGTTCGTTG	4369bp	Skeleton plasmid fragment Ⅱ, using pUt-310Ⅳ as template
P2-311Ⅳ-R	ACCGCATAGGGTAATAACTGATA		
AgAS-312Ⅳ-F	TTCGGTTAGAGCGGATCTAFGGCAACTGGTTGGAAG	2650bp	Synthetic *AgAS*
AgAS-312Ⅳ-R	CGGTCAACGAACTATAATTAACTAAACATGGCCATGCCTTCCTCCT		
P1-312Ⅳ-F	TATTACCCTATGCGGTGTGAAATA	3344bp	Skeleton plasmid fragment Ⅰ, using pUt-311Ⅳas template
P1-312Ⅳ-R	ATCCGCTCTAACCGAAAAGGAAGG		
P2-312Ⅳ-F	TATAGTTCGTTGACCGTATATTCT	4347bp	Skeleton plasmid fragment Ⅱ, using pUt-311Ⅳ as template
P2-312Ⅳ-R	ACCGCATAGGGTAATAACTGATA		
UP14-F	CGAAAAACAGGCCACTAAACTGTGGCGCTTTTTAA	547bp	Upstream homologous arm of *ARS1414a* site.
UP14-R	TGGCCTTTATGGCCTGTGGTATGTAAACGGATGG		
Down14-F	ACAGGCCATAAAGGCCACCCTTAATGACCAGTAAGG	513bp	Downstream homologous arm of *ARS1414a* site.
Down14-R	TGCTATCAACATCGGATAATTTGAGCAATGAT		
85C214-F	TCCGATGTTGATAGCAGCTTTTTCC	2292bp	Synthetic *SrUGT85C2* with its promoter and terminator
85C214-R	CCTTACTCACCATATATATATATATATATATG		
74G114-F	TATATGGTGAGTAAGGAAAGAGTGAG	2283bp	Synthetic *SrUGT74G1* with its promoter and terminator
74G114-R	AATGACCGGTAGAGGTGTGGTCAATA		
91D2e14-F	ACCTCTACCGGTCATTATCAATACTGCCATTT	2152bp	Synthetic *SrUGT91D2* with its promoter and terminator
91D2e14-R	ACCTCTACCGGTCATTATCAATACTGCCATTT		
P14-F	CGACGGTATCGATAAATTGAATTGAATTGA	3679bp	Skeleton plasmid fragment, used pUSC02 as template
P14-R	AGTGGCCTGTTTTTCGACATAA		
74G1MU2-1F	GATTATAGTTCGTTGACCGTATAT	1537bp	A bidirectional promotor *TEF2*

74G1MU2-1R	GTTGCCAAATACGCTTCTCCTGCAC		and *PGK1*, and *SrUGT74G1* upstream sequence , 3' end contains mutants that bases of underline
74G1MU2-2F	GAAGCGTATTTGGCAACATTCAAAC	1149bp	*SrUGT74G1* downstream sequence , 5' end contains mutants that bases of underline, and 3' end contains linker sequence
74G1MU2-2R	CATAGAACCACCACCAGCCTTAATTAGCTCACTTA		
85C2U2-F	TGGTGGTGGTTCTATGGATGCAATGGCTACAACTG	2753bp	*SrUGT85C2* with its terminator, and , and the UP and Downstream arm of *ARS1414a* site , used pU-UGT as template
85C2U2-R	ATGTCGAAAAACAGGCCACTAAAC		
91D2eU2-F	GAACCACCACCTTAACTCTCATGATCGATGGC	1488bp	Synthetic *SrUGT91D2e_NO.5*,
91D2eU2-R	CGGTCAACGAACTATAATCATGTACAACGTTACTTATCA		
PU2-F	GCCTGTTTTTCGACATAAATGATG	3695bp	Skeleton plasmid fragment , using p14-UGT as template
PU2-R	GTTAAGGTGGTGGTTCTGGTCGACG		
Up16-F	CGAAAAACAGGCCACTGGATATACCAAAAATTCTCT	498bp	Upstream homologous arm of *ARS1622b* site.
Up16-R	AGGCCTTTATGGCCTTTATTGGAGTTATTGGATA		
Down16-F	AAGGCCATAAAGGCCTCGAAAGGAGAAGTAATGGTA	490bp	Downstream homologous arm of *ARS1622b* site.
Downd-R	TGAAGTCTTTGCCAACAATCGAA		
ProT16-F	GTTGGCAAAGACTTCATTATCAATACTGCCATT	688bp	promoter *TDH3*
ProT16-R	GTGGACATTTGTTTGTTTATGTGTGTTT		
UGP16-F	CAAACAAATGTCCACTAAGAAGCACAC	1517bp	*UGP1*, amplified from *S.cerevisiae* genome
UGP16-R	CAATCTCGAGTCAATGTTCCAAGATTTGCA		
PUGP16-F	CATTGACTCGAGATTGAATTGAATTGAAATCG	3676bp	Skeleton plasmid fragment , using pUSC01 as template
PUGP16-R	AGTGGCCTGTTTTTCGACATAA		
HXK1HP-F	ATTACTCTTAAGCGCCAATGATACCAA	1465bp	*HXK1*, amplified from *S.cerevisiae* genome
HXK1HP-R	ATGGTTCATTTAGGTCCAAAGA		
ProHP-F	GGACCTAAATGAACCATTTTAGTTAATTATAGTTCGT	1310bp	A bidirectional promotor *TEF2* and *PGK1*
ProHP-R	GACATCTCGAGTTGTTTTATATTTGTTGTAA		
PGM2HP-F	AACAACTCGAGATGTCATTTCAAATTGAAAC	1726bp	*PGM2*, amplified from *S.cerevisiae* genome
PGM2HP-R	TTCGCTTAAGTACGAACCGTTGGTTC		
PHP-F	GTTCGTACTTAAGCGAATTTCTTATGATTTATGA	3845bp	Skeleton plasmid fragment , using pUSC02 as template
PHP-R	GGCGCTTAAGAGTAATAATTATTGCTTCCA		
HP-F	CTATGTTGATAGCAGCTTTTTCCATT	4923bp	amplified *HXK1* and *PGM2* from *S.cerevisiae* genome, using p02-HD as template
HP-R	GGCAGTATTGATAATGACCGGTAGAGGTGTGGTCAATA		
PUDP1-F	GGTCATTATCAATACTGCCATTTC	3674bp	Skeleton plasmid fragment Ⅰ ,

PUDP1-R	ACCGCATAGGGTAATAACTGATA		using p01-UGP as template
PUDP2-F	TATTACCCTATGCGGTGTGAAATA	3148bp	Skeleton plasmid fragment Ⅱ, using p01-UGP as template
PUDP2-R	GCTGCTATCAACATAGTCTTTGCCAACAATCGAA		

Table S4. The primers used for detection in this study.

integration site	primers name	seqence
	1309-F	TCTTTGGAACGCCATAACCAA
ARS1309a	1309-R	CCCTTGAATTCGTATTTATGTA
	YPL-F	CCCGCTCGATTTGATTCATGAA
YPL062w	YPL-R	TTTGTCGGCATAGGCAGCAGC
	erg9-F	GTCCGGCGGTTTCAAATAATCT
P*erg9*	erg9-R	AATCAAACGGGTCAAACCATCA
	YJL-F	CTCCAAGATCTCTGAAGTTG
YJL064w	YJL-R	TAAAGAGGCTTCCAGGGTCG
	delta15-F	GAACGAAAGAATTTTACCACTTTGTA
YPLdelta15	delta15-R	GTCTAAAAAAGAAAGCTCGCACTCAG
	1414-F	CGTCCGTCTTTGAAGTTATATA
ARS1414a	1414-R	TTTGTAATGTCCACGACTTATC
	1622-F	AAGCCCCAAAAATATTATAGGG
ARS1622b	1622-R	GTTCCTCAGATGATAGTTGG

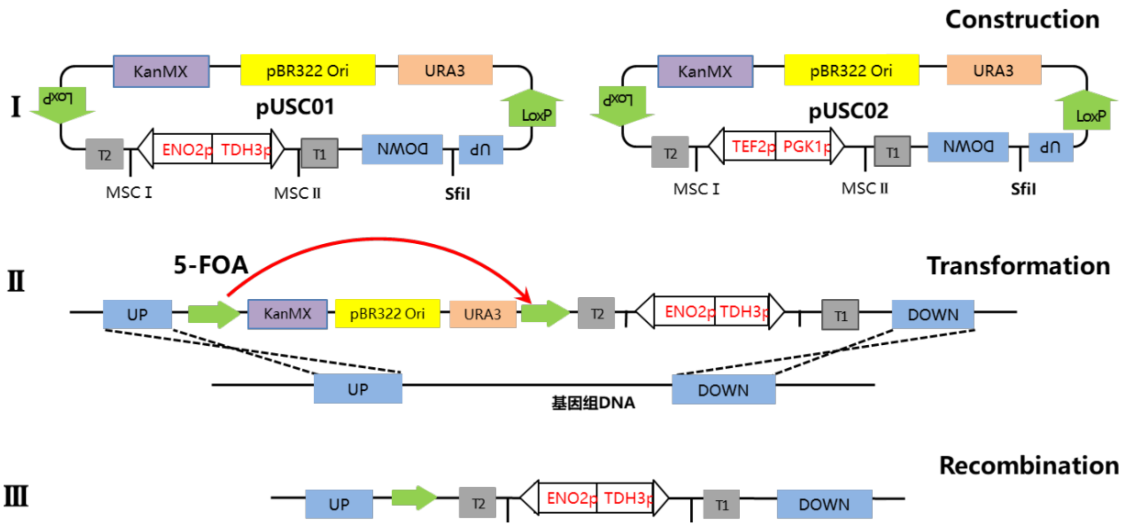

Figure S1. Schematic diagram of homologous recombinant plasmid operation . Step Ⅰ, The genes inserted into MSCⅠ/MSCⅡ site of pUSC01/pUSC02. Step Ⅱ, Digested the constructed plasmid with restriction enzyme SfiI which between the up and downstresm arm, transformed into *Saccharomyces cerevisiae*; the sequence of up and downstresm homologous arm homologous recombined with chromosome. Step Ⅲ, Two codirectional LoxP sequences were recombined without screening pressure, thus the intermediate marker is lost.

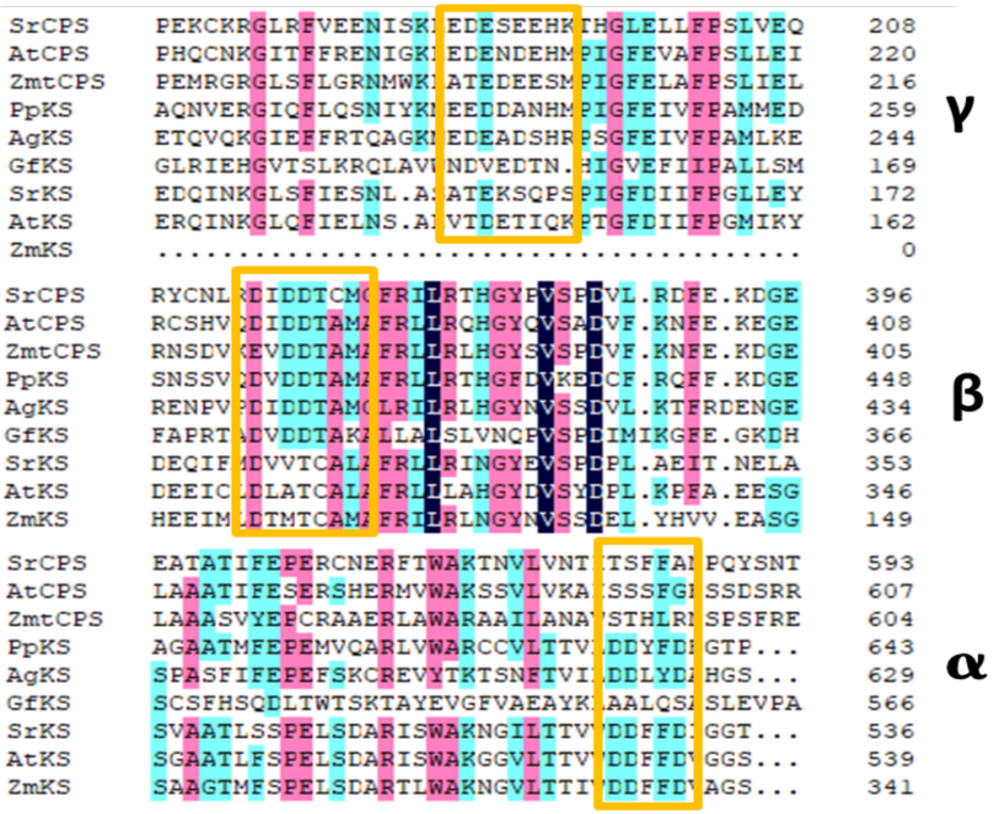

Figure S2. Homologous sequence alignment of diterpene synthase. The conserved motif "DXDD" was found in the β domain of class II diterpene synthases SrCPS, AtCPS, ZmtCPS and bifunctory diterpene synthases PpKS, AgAS and GfKS. The α domain of class I diterpene synthases AtKS, SrKS, ZmKS and bifunction diterpene synthases PpKS and AgAS also contains conserved motif DDXXD, while the α domain of GfKS is conserved motif DEYME.

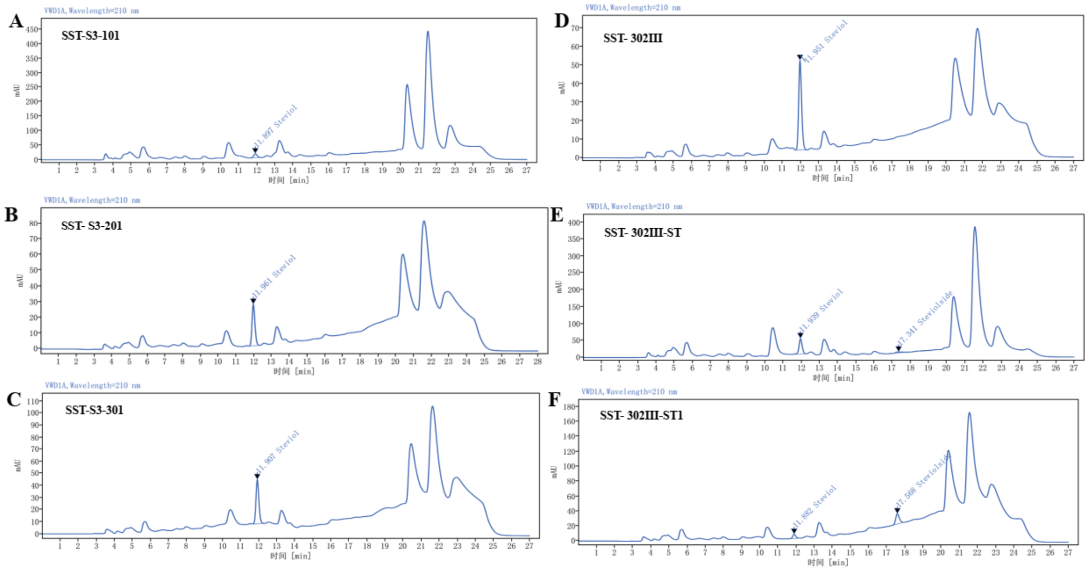

Figure S3. The Liquid chromatogram of fermentation product of recombinant strain. A-F were strains SST-101, SST-201, SST-301, SST-302Ⅲ, SST-302Ⅲ-ST and SST-302Ⅲ-ST1, Respectively.
